# Peritoneal Hydatidosis: Report of an Exceptional Case

**DOI:** 10.7759/cureus.117437

**Published:** 2026-10-04

**Authors:** Mahmoud Dabbagh, Amine Maazouz, Hakim El Kaoui, Sidi Mohammed Bouchentouf, Mountassir Moujahid

**Affiliations:** 1 Surgery, Mohammed V Military Training Hospital, Rabat, MAR

**Keywords:** abdominal surgery, albendazole, disseminated hydatid cyst, echinococcus granulosus, peritoneal hydatidosis

## Abstract

Hydatidosis is a cosmopolitan parasitosis that poses a major public health risk in endemic regions such as Morocco. Secondary implantation of *Echinococcus granulosus* larvae in the peritoneum is called peritoneal hydatidosis. This condition presents itself in a polymorphic manner, and its detection requires a combination of various aspects, including epidemiological, clinical, biological, and radiological elements.

We report an uncommon case of peritoneal hydatidosis in a patient with a history of surgically treated hepatic hydatid disease, who presented with progressive abdominal pain and distension. Physical examination was unremarkable, whereas hydatid serology was strongly positive. CT was essential for establishing the diagnosis by demonstrating both hepatic and peritoneal cystic lesions.

The patient had surgery to remove the cyst. Post-operative recovery was uneventful, and albendazole treatment was initiated as adjuvant therapy.

Our case report underscores the challenges faced in diagnosing this uncommon condition, the significance of diagnostic imaging in achieving an accurate diagnosis, and the advantages of surgical intervention.

## Introduction

Peritoneal hydatidosis is an uncommon and potentially serious manifestation of hydatid disease, representing a small proportion of intra-abdominal localizations [1]. It is most often secondary to rupture of a primary hepatic or splenic hydatid cyst, which may be spontaneous, traumatic, or iatrogenic. In rare cases, it may occur as a primary localization [1].

Despite advances in imaging techniques, particularly CT, the diagnosis remains challenging due to its nonspecific clinical presentation, which may mimic other causes of abdominal pathology [2]. Consequently, it is frequently diagnosed at an advanced stage or discovered intraoperatively.

Management is mainly surgical and aims at the complete removal of cysts while preventing peritoneal dissemination. Adjunctive medical therapy with albendazole plays a key role in reducing recurrence risk. However, optimal management strategies remain debated, especially in disseminated forms [3].

We report an exceptional case of peritoneal hydatidosis revealed by chronic abdominal symptoms, which posed a diagnostic challenge. Through this observation, we aim to highlight the clinical features, diagnostic approach, and therapeutic management of this rare condition.

## Case presentation

A 56-year-old man from an endemic rural area presented with progressive abdominal pain and distension that had developed over several months. He had a history of treated hepatic hydatid disease. The patient denied any recent history of trauma.

On admission, the general condition was preserved. Vital signs were stable, with no fever. Physical examination revealed a moderately distended abdomen with diffuse tenderness, without signs of peritoneal irritation. No palpable mass was clearly identified.

Laboratory investigations showed no major abnormalities. Hydatid serology was positive, with a value of 30 U (reference range: 0-8 U) (Table 1).

**Table 1:** Laboratory findings of the patient

Parameter	Result	Reference Range
White blood cells count (10^3^/uL)	10	4.5 to 11.0
Creatinine (mg/L)	10	6 to 13
Urea (g/L)	0.22	0.15 to 0.38
Hemoglobin (g/dL)	14.1	13 to 17
Hydatid serology (U)	30	0-8

Abdominal imaging, particularly contrast-enhanced CT scan, revealed a subhepatic peritoneal hydatid cyst measuring 50 × 38 mm (Figure 1a), associated with a hepatic cyst suggestive of hydatid origin (Figure 1b), without intraperitoneal fluid or collection. These findings were consistent with disseminated peritoneal hydatidosis.

**Figure 1:**
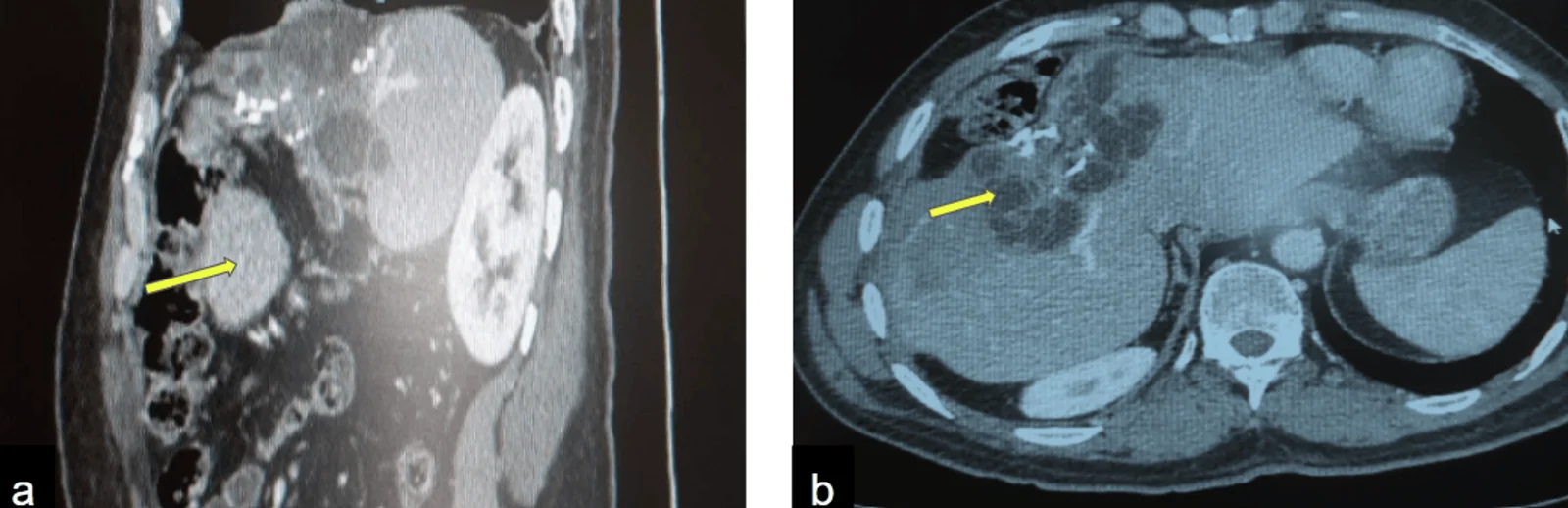
CT scan findings a: Sagittal abdominal CT scan showing a subhepatic cystic lesion (yellow arrow); b: Axial CT scan showing multiple hepatic hydatid xysts (yellow arrow)

The patient underwent an exploratory laparotomy, which revealed a subhepatic cystic mass adherent to the right colic flexure (Figure 2a). Mobilization of the right colon was challenging, allowing subsequent cyst resection with complete removal of the germinative membrane (Figures 2b, 3). Multiple pseudomembranes were observed over the small bowel loops (Figure 2c), along with daughter cysts interspersed between the intestinal loops. Followed by extensive peritoneal lavage using a scolicidal solution, and two Redon drains were placed in the subhepatic space and pouch of Douglas before layered closure.

**Figure 2:**
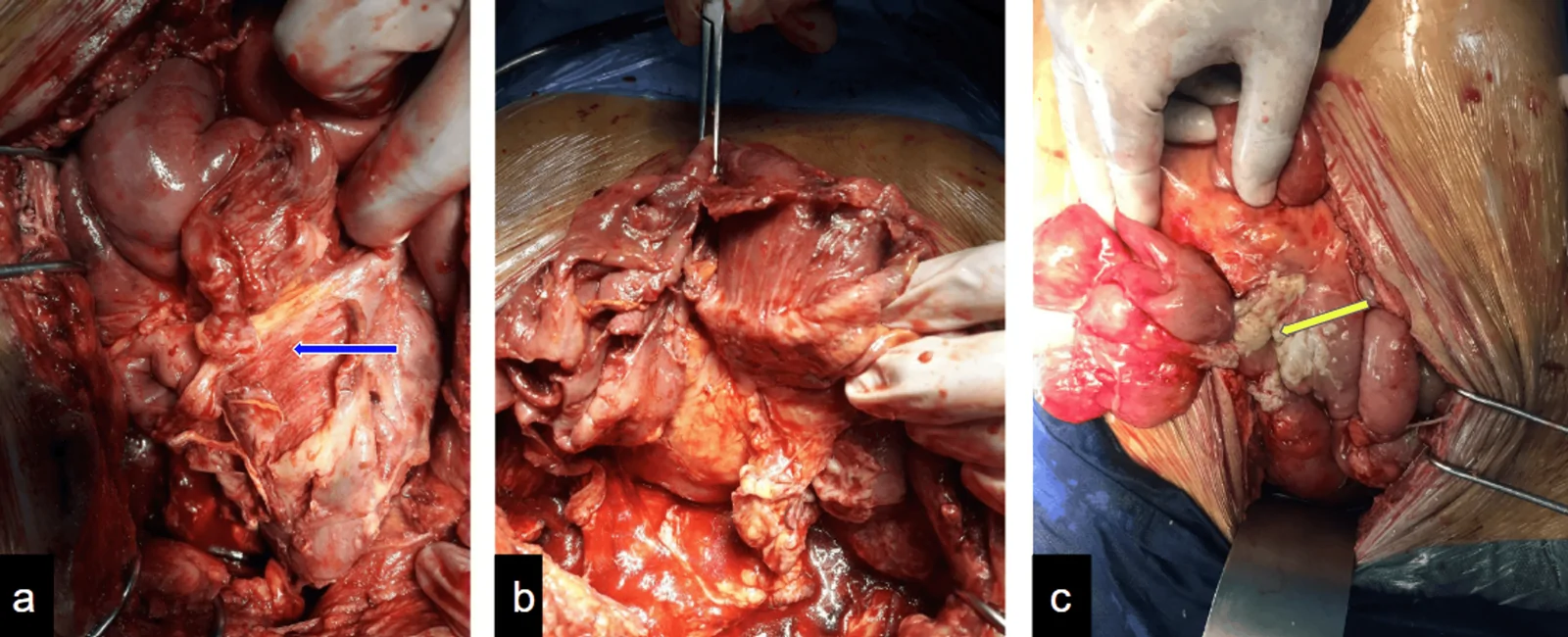
Intraoperative views a: Subhepatic cystic mass adherent to the right colic flexure (blue arrow); b: Post-removal view of the peritoneal hydatid cyst; c: Intraoperative image of the pseudomembrane (yellow arrow)

**Figure 3:**
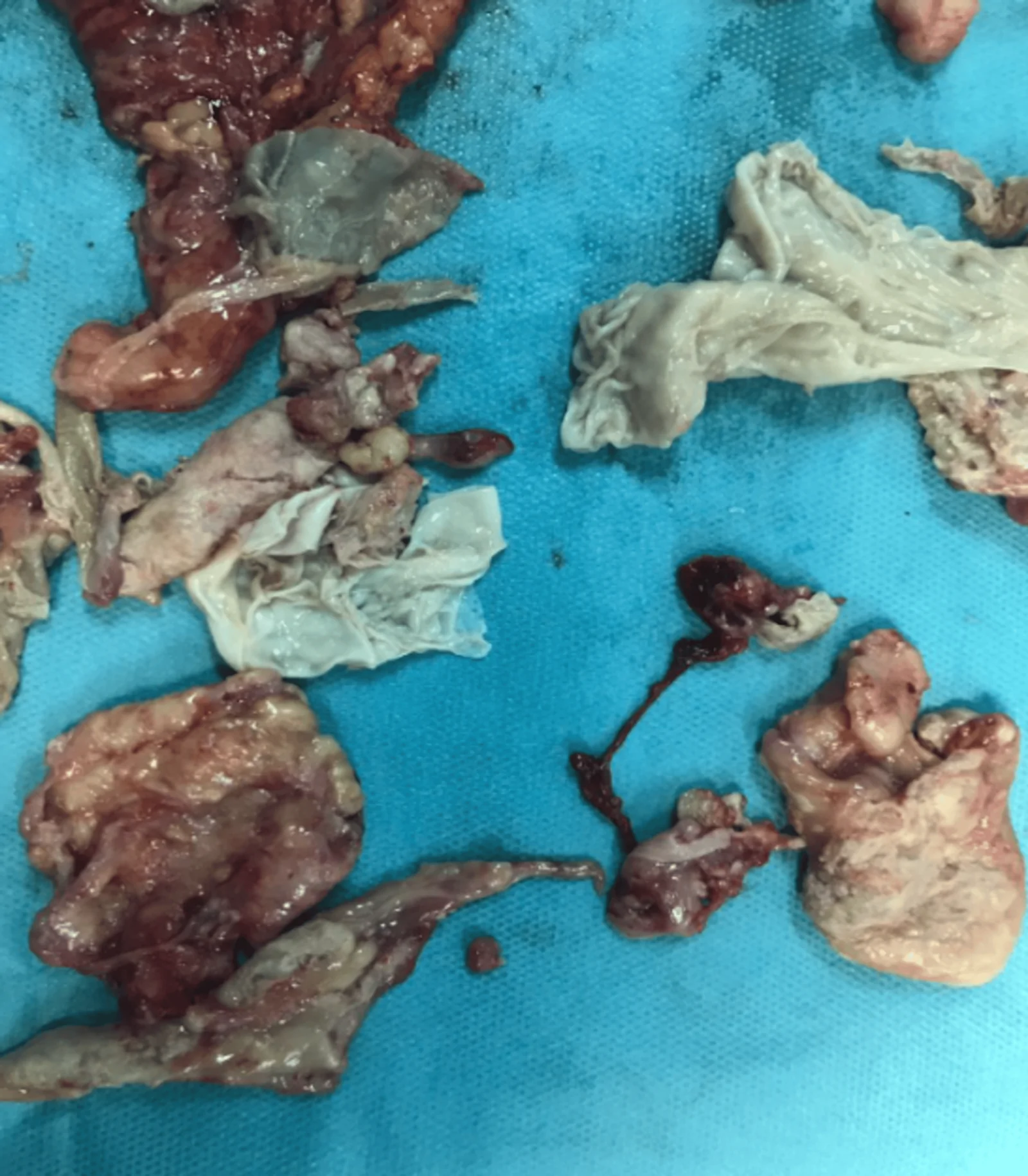
Post-operative view showing multiple germinative membranes

The post-operative course was uneventful, with favorable clinical and biological evolution. The patient received albendazole 400 mg twice daily for 10 days, followed by a 10-day interruption, with this cycle continued for a total duration of three months.

The patient was followed up after surgery and remained asymptomatic, with no clinical evidence of recurrence. No follow-up CT or ultrasound or repeat serology was performed during this period.

## Discussion

Peritoneal hydatidosis is a rare but serious complication of hydatid disease, representing approximately 5-16% of abdominal localizations [4]. It is most frequently secondary to rupture of a hepatic hydatid cyst, which may be spontaneous in the majority of cases or triggered by trauma or surgical manipulation [4].

The pathophysiology involves the dissemination of protoscolices into the peritoneal cavity, leading to the implantation and development of multiple cysts. This mechanism explains the frequent occurrence of diffuse forms with numerous lesions, sometimes exceeding one hundred cysts, as reported in the literature [5].

Clinically, the disease evolves insidiously, often remaining asymptomatic for years. When present, symptoms are nonspecific and related to mass effect or complications such as rupture, infection, or compression of adjacent organs [5].

In our case, the history of previously treated hepatic hydatid disease supports a secondary peritoneal origin. Although CT demonstrated a subhepatic peritoneal cyst associated with a hepatic cyst, surgical exploration revealed more extensive peritoneal involvement, with pseudomembranes and daughter cysts distributed between the intestinal loops. The adherence of the subhepatic cyst to the right colic flexure made mobilization technically challenging. This observation highlights the potential discrepancy between the extent of peritoneal disease suggested by preoperative imaging and the actual findings at laparotomy, particularly in patients with a history of hepatic hydatid disease.

Imaging plays a pivotal role in diagnosis. Ultrasonography is useful as a first-line tool, but the CT scan remains the gold standard, allowing precise assessment of the number, size, and distribution of cysts, as well as detection of associated visceral involvement [6].

Therapeutically, surgery remains the cornerstone of treatment, especially in disseminated forms. The objective is maximal removal of cysts while preventing spillage and recurrence. This is often combined with peritoneal lavage using scolicidal agents. Medical treatment with albendazole is essential as an adjunct to reduce recurrence risk and manage residual disease [7].

Despite advances in management, recurrence remains the main concern, particularly in extensive or late-diagnosed forms [8].

## Conclusions

Peritoneal hydatidosis is an uncommon and often secondary localization of hydatid disease. Its diagnosis relies heavily on imaging, particularly a CT scan. Surgical management combined with antiparasitic therapy remains the standard of care. Early diagnosis and adequate treatment of primary hydatid cysts are essential to prevent this severe complication.
